# SeptAsTERS- SeptiCyte® RAPID as assessment tool for early recognition of sepsis – a prospective observational study

**DOI:** 10.1007/s15010-024-02409-4

**Published:** 2024-11-22

**Authors:** M. von der Forst, L. Back, K.M. Tourelle, D. Gruneberg, M.A. Weigand, F.C.F. Schmitt, Maximilian Dietrich

**Affiliations:** Medical Faculty Heidelberg, Department of Anesthesiology, University Hospital Heidelberg, Im Neuenheimer Feld 420, 69120 Heidelberg, Germany

**Keywords:** Sepsis, Point-of-care systems, Host gene response, Intensive care, Blood culture, Diagnostic microbiology

## Abstract

**Purpose:**

Early recognition of sepsis is critical to patient outcome, with mortality increasing with every hour of delay in treatment. The aim of this study was to investigate the use of a point-of-care molecular host response assay to differentiate sepsis from inflammation after surgery.

**Methods:**

Three molecular host response assays (SeptiCyte® RAPID) were performed in 61 patients after major abdominal surgery with admission to the intensive care unit and drawn blood cultures. The first (T0) was taken ± 3 h around the time of obtaining blood cultures, the second 24 h later (T24) and the third at discharge from the intensive care unit (Tex). The primary endpoint was the agreement of SeptiCyte® RAPID results with the diagnosis of sepsis. SeptiScore® indicates sepsis probability (low risk 0 - high risk 15). Patients were retrospectively classified into sepsis and inflammation by three blinded experts.

**Results:**

25 (42.4%) patients were categorized as “inflammation” and 34 (57.6%) patients as “sepsis”. At T0 and T24 septic patients showed significantly higher mean SeptiScores® of 8.0 (± 2.2 SD) vs. 6.3 (± 2.1 SD) and 8.5 (± 2.1 SD) vs. 6.2 (± 1.8 SD), respectively. The Receiver Operating Curves (ROC) for the ability to discriminate between sepsis and inflammation had an Area Under the Curve (AUC) of 0.71 (T0) and 0.80 (T24).

**Conclusion:**

Embedded in a comprehensive diagnostic algorithm molecular host response analysis could broaden the possibilities for infection diagnostics to differentiate between sepsis and inflammatory response after surgery. But validation in larger cohorts is needed.

## Introduction

Despite a sharp decline in mortality from 1990 to 2017, sepsis is still one of the leading causes of death worldwide [1]. In sepsis, an inadequate host response to an infection leads to life-threatening (multi-) organ failure, often accompanied by progressive circulatory failure (septic shock) [2, 3]. Established protocols for the treatment of suspected sepsis have been in place for many years and a large number of research activities are aimed at gaining new insights into this complex clinical syndrome. However, despite all these efforts, sepsis-associated mortality is currently still between 20 and 30% [4, 5]. The globally harmonized treatment guidelines for sepsis therapy are regularly updated and the most important steps in the initial treatment of sepsis are summarized in a so-called “one-hour” bundle. This includes the rapid initiation of empirical broad-spectrum antibiotic therapy in combination with prior collection of samples - primarily blood cultures - for diagnostic microbiology. In addition, infection source control is crucial for causal treatment, while supportive intensive therapy- including organ replacement procedures - is often required [6]. Especially under perioperative circumstances diagnosis of infections and sepsis is a challenge even for experienced physicians. Traditional host biomarkers such as C-reactive Protein (CRP) or procalcitonin (PCT) are of limited use in the setting of surgery induced inflammatory response, drug- or surgery-induced immunodeficiency, underlying malignancy or high levels of multimorbidity. Further in about one third of patients with sepsis, no causative pathogen can be identified [7].

Early detection of sepsis is therefore crucial for patient outcome and with mortality increasing with every hour of delay in treatment, there is an urgent clinical need for methods to diagnose this condition more rapidly [8]. But even the exclusion of an infection has far-reaching consequences, such as the recommendation to stop antimicrobial therapy immediately [3]. In one of its statements, the Surviving Sepsis Campaign 2021 takes these factors into account by recommending “…using a performance improvement program for sepsis, including sepsis screening for acutely ill, high-risk patients…” [6].

The hypothesis of the underlying SeptiCyte® RAPID as Assessment Tool for Early Recognition of Sepsis (SeptAsTERS) study was that the use of a point-of-care molecular host response assay could be beneficial in a surgical patient population to better differentiate between sepsis and other causes of inflammation (e.g. surgery alone). By eliminating time-consuming uncertainties, this could lead to an improvement in treatment in the early phase of sepsis and help to avoid unnecessary therapies, e.g. the administration of antibiotics for non-infectious diseases.

## Methods

### Study design

SeptAsTERS is a prospective observational study. Due to the pilot nature of the study, it was not possible to perform a sample size calculation or power analysis. Inclusion in the study did not affect patients’ treatment, as the results of the point-of-care molecular host response assay were not made available to the treating physicians. This study was conducted at the surgical intensive care unit (ICU) at the Department of Anesthesiology of Heidelberg University Hospital in Germany.

The study has been conducted in accordance with the Declaration of Helsinki and the current version of the Professional Code of Conduct for Physicians of the Baden-Württemberg Medical Association. The study was approved by the Ethics Committee of the Medical Faculty of Heidelberg University (S-118/2021) and was registered in the German Clinical Trials Register (DRKS00024891) prior to enrolment.

### Patient recruitment

The recruitment period for the SeptAsTERS study took place from May 2021 until July 2023. Potential patients were screened daily during the regular working hours by the study team. Participants underwent major abdominal surgery and experienced clinical deterioration during their clinical stay. If this further implicated the need of intensive care therapy and the treating physician decided to draw blood cultures for diagnostic as part of the standard therapy the patient became potentially eligible for the study. For an enrolment in the study the following inclusion and exclusion criteria were defined:

#### Inclusion criteria

- Age ≥ 18 years.
- Patients underwent major elective abdominal surgery.
- First clinical deterioration with suspected infection and admission to the intensive care unit.
- Need to take blood cultures as a part of standard therapy.

#### Exclusion criteria

- Pregnancy.
- Inclusion in another interventional study.
- Refusal to participate in the study.

**Fig. 1:**
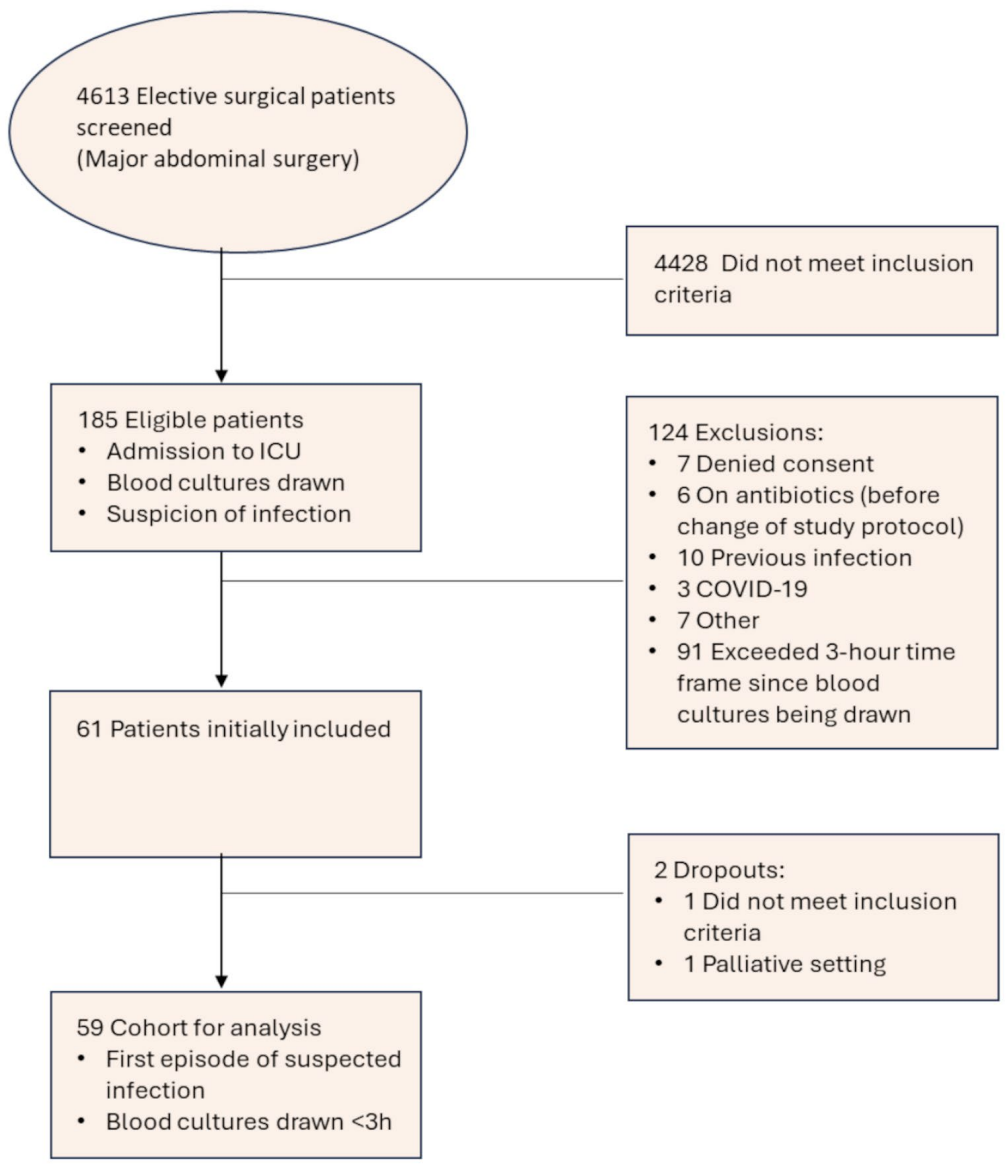
Overview of screening and study recruitment

In total more than 4600 patients which underwent elective major abdominal surgery were screened, most of them did not meet the inclusion criteria because there was no suspicion of infection. Out of these 185 patients were eligible, but many had further exclusion criteria (Fig. 1). Finally, 61 patients were enrolled. One patient did not fulfil study criteria in a second survey and in one other case the therapy setting was switched to best supportive care after the first hours, so these patients dropped-out after the first measurement following the study protocol. For final analysis *n* = 59 patients were available.

Most patients with a suspicion of sepsis included into the study provided written informed consent prior to enrolment. Due to the severity of the clinical condition, patients with sepsis in part lack decision-making capacity. Considering Declaration of Helsinki [9], this condition constitutes a fundamental characteristic of the patient cohort under investigation. In cases where patients were unable to provide consent, legal support was arranged, and consent forms were signed by their relatives or legal representatives. Hence, our ICU has a standardized procedure for patients unable to provide consent independently, facilitated either by their relatives or legal representatives. In certain instances, a legal representative may not yet be available or may be unreachable in acute situations. To address this circumstance, a multi-stage inclusion process, following the “Giessener Model”, has been implemented [10].

### Blood sample collection and SeptiCyte® RAPID measurements

For a simultaneous testing of potentially septic patients which were enrolled into the study, the protocol provides the collection of three blood samples. The first (T0) was taken ± 3 h after admission around the time of obtaining blood cultures, the second 24 h later (T24) and the third on the day of discharge from intensive care (Tex). In addition, all available clinical and laboratory parameters were recorded at the same time as the blood samples were taken. For the final analysis the whole medical record (microbiological findings, antibiotics, further operations, diagnostic procedures, etc.) beginning from seven days before the enrolment until 6 weeks after was taken into consideration.

The SeptiCyte® RAPID Test based on the Idylla System™ (Biocartis NV, Mechelen, Belgium) is an approved and CE certified assay. The application of SeptiCyte® RAPID was conducted according to the standardized protocols provided by the manufacturer. Venous or arterial blood samples were collected from participants, with 2.5 ml of blood transferred into specialized PAXgene tubes. The samples were transported to the laboratory and mostly processed immediately after collection.

The measurement procedure involves transferring 900 μl of PAXgene-stabilized blood into a specialized SeptiCyte® RAPID cartridge to automatically perform an RT-qPCR for the detection and relative quantification of the two PLAC8 and PLA2G7 mRNA targets. The SeptiCyte® RAPID test, which runs on the Biocartis Idylla System, has been shown to have a high correlation with the validated SeptiScore® values generated by the original Septicyte® LAB test [11, 12]. Both mRNA Targets are results of an analysis of different Septicyte® LAB biomarkers (LAMP1, CEACAM4, PLA2G7, PLAC8) and showed the most promising results for the discrimination between sepsis and sterile inflammation [13, 14].

**Fig. 2:**
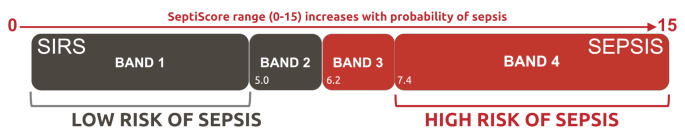
SeptiScore® range with corresponding bands

The analysis of expression levels was performed quantitatively using a validated algorithm that considers the expression levels of the aforementioned biomarkers [13]. The interpretation of results was based on predefined thresholds provided by SeptiCyte® RAPID to determine the presence of sepsis. Test results are automatically calculated and presented through a digital report. As a result of the test, the software generates a SeptiScore®, ranging from 0 to 15. SeptiScore® is computed as the difference between the RT-qPCR cycle of quantification values for PLA2G7 and PLAC8 and is proportional to the probability of sepsis. The SeptiScore® is interpreted using four discrete interpretation bands that correspond to increasing likelihood of sepsis (Fig. 2).

The SeptiScore® has demonstrated the ability to accurately differentiate sepsis from non-infectious systemic inflammatory response syndrome (SIRS) in clinical studies [12, 13]. To ensure the reproducibility of results, all steps of the analysis were carried out with strict adherence to the prescribed procedures.

### Research question and endpoint criteria

This study aimed to investigate the utility of bedside measurement of the molecular host response to an infectious agent using the point-of-care test SeptiCyte® RAPID. The present research seeked to address the following questions: Do the results of bedside testing of sepsis probability provide consistent findings compared to established clinical parameters and microbiological analysis, particularly blood cultures? Can bedside determination of the molecular host response lead to a temporal improvement in diagnosis compared to conventional clinical procedures? Is there a correlation between the severity of infection and the molecular host response?

To answer these research questions the following endpoints have been defined for the underlying analysis:

#### Major

Agreement of SeptiCyte® RAPID results with the clinical diagnosis of sepsis (retrospectively by blinded experts).

#### Minor

- Agreement of SeptiCyte® RAPID results with blood cultures taken at the same time (max. ±3 h).
- Comparison of SeptiCyte® RAPID results with all other microbiological findings.
- Comparison of SeptiCyte® RAPID results with routine clinical infection parameters (e.g. PCT, CRP, Leukocyte Count).

### Post hoc-diagnosis

For the determination of the primary endpoint a post-hoc assessment of all patients by three independent intensive care professionals, which were not involved into the study, was performed. The process followed a model similar to the one described in the online data supplement Part 3 by Miller et al. [13] based on FDA Guidance and Publications by Klein Klouwenberg et al. [15–17].

The three experts did not know about each other. Everyone got the same sheet with the study number and the following clinical information at T0, T24 and Tex: Age, gender, primary surgery, inclusion on postoperative day, ICU length of stay, total hospital length of stay, APACHE-II score, SOFA score, Glasgow Coma Scale, body temperature, vasoactive inotropic score, mean arterial pressure, heart rate, respiratory rate, Horovitz quotient, Leukocytes, SIRS criteria, oxygen demand, creatinine, bilirubin (total), CRP, platelets, pH value (ABG), hemoglobin, glucose, lactate, microbiological findings, antibiotic therapy, death during hospitalization, radiological findings and interventions.

**Fig. 3:**
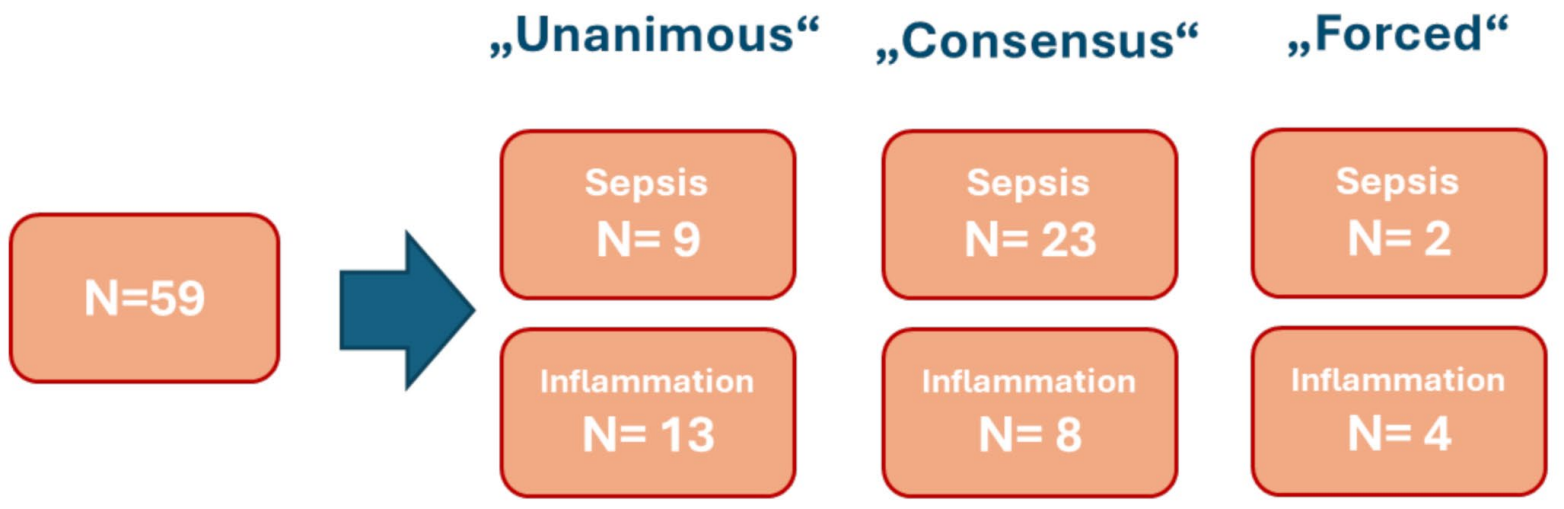
Total number of patients divided into “Sepsis” and “Inflammation” corresponding to post-hoc diagnosis results

For the classification process, each expert independently utilized the provided information to designate a status of sterile inflammation, sepsis, or indeterminate. A classification was deemed “unanimous” if all three expert panelists categorized a patient as either sterile inflammation or sepsis. If a “consensus” (majority decision) was achieved on a classification, it was adopted. In cases where a subject received one vote each for sterile inflammation, sepsis, and indeterminate, it was categorized as indeterminate. Subsequently, indeterminate subjects underwent a “forced” classification into either sterile inflammation or sepsis during a second review conducted by the expert which chose indeterminate. The diagnosis results were “Unanimous” in 37.3%, “Consensus” in 52.5% and “Forced” in 10.2%. The detailed results of post-hoc diagnosis are shown in Fig. 3.

### Statistical analysis

Statistical data were collected with an electronic database system (Microsoft Excel®, Microsoft Deutschland GmbH, Unterschleißheim, Germany). The statistical analysis and figures were performed with SPSS (Statistical Product and Services Solutions, Version 25, SPSS Inc., Chicago, IL, USA) and Graphpad Prism (Version IX, GraphPad Software, La Jolla, USA). Descriptive statistics were carried out for the complete dataset. Missing values were not imputed because of the small sample size. The Student`s t-test and one-way ANOVA with Kruskal-Wallis, as well as the Dunns-Multiple Comparison and Mann-Whitney-U Test, were used for the determination of significance (*=*p* < 0.05, **=*p* < 0.01, ***=*p* < 0.001, ****=*p* < 0.0001). The study fulfills the requirements of the STROBE statement [18].

As authors utilizing Generative AI and AI-assisted technologies we recognize the ethical responsibilities associated with these tools. In accordance with best practices and principles of scientific integrity, we hereby declare that during the preparation of this work, the authors utilized ChatGPT 3.5 (OpenAI, San Francisco, USA) and the Perplexity (https://www.perplexity.ai) program to revise grammar, enhance language coherence, and conduct research on scientific content. Following the utilization of these tools, the authors meticulously reviewed and edited the content to ensure its compliance with the requirements. Consequently, the authors assume full responsibility for the integrity and accuracy of the publication.

## Results

### Patient characteristics

The characteristics of the analyzed patients are shown in Table 1, divided into the groups inflammation and sepsis according to the post-hoc diagnosis. The sepsis group contained more male individuals (70.6% vs. 56%) than the inflammation group. Most of the patients were categorized ASA II (20%) or III (78%) preoperatively and the major part of surgeries were gastrointestinal (42.4%) or pancreatic (33.9%) high-risk procedures. The indications for surgery were predominantly malignancies (*n* = 45), but also chronic inflammatory bowel disease (*n* = 4), bariatric surgery (*n* = 3), pancreatitis (*n* = 3) and others (*n* = 4). The lCU length of stay was in a median range of 6 days, whereas the mean was much higher for the inflammation group (22.5 vs. 8.9 days) with one outlier in this group having an ICU length of stay of 303 days. Also, the total length of stay was longer for the inflammation group, even after leaving out the outlier (length of stay 338 days) with a mean of 46.0 vs. 43.8 days, this difference was not significant, further it is to take into consideration that the 28-day mortality was higher in the sepsis group (*n* = 6 vs. *n* = 0) which shortens the length of stay. The in-hospital mortality was higher in the sepsis group (26.5% vs. 8%), but regarding the small sample size this difference did not become statistically significant.

**Table 1:** Patient baseline characteristics

	Inflammation (*n* = 25)	Sepsis (*n* = 34)
Age (years) [Median, Min-Max]	67 (30–82)	68 (30–84)
Sex (m/w)	14/11	24/10
BMI (kg/m^2^) [Median, Min-Max]	26.7 (15.0-69.2)	26.4 (16.4–45.8)
ASA Classification	ASA 2: 5 ASA 3: 20	ASA 2: 7 ASA 3: 26 ASA 4: 1
Kind of primary surgery	Gastrointestinal: 12 Pancreatic: 8 Liver: 1 Vascular: 1 Other: 3	Gastrointestinal: 13 Pancreatic: 12 Liver: 5 Vascular: 1 Other: 3
Length of stay ICU (d) [Median, Min-Max]	6 (1-303)	6 (0–31)
Length of hospital stay (d) [Median, Min-Max]	40.0 (11–338)	36.5 (1-133)
28-Day Mortality	n = 0	n = 6
In hospital Mortality	n = 2	n = 9

The comparison of patient scores as well as different laboratory parameters at the timepoint of enrolment are shown in Table 2. The patients of the “Inflammation” and the “Sepsis” groups were included into the study at a median of 6 and 7 days postoperatively, respectively. In total 13 patients were included during the first three postoperative days, more than the half of all patients (*n* = 34) during the first week after and 85% (*n* = 50) until the end of week two after surgery. The data show that especially regarding SOFA and Apache II Score, the sepsis group had a significantly higher illness severity (Table 2). The vasoactive inotropic score and the lactate levels were also higher in sepsis patients. A comparison of the standard infection parameters leucocytes, CRP and PCT (*p* < 0.05) revealed that only the latter exhibited a significant difference between the two groups. The former two demonstrated a trend towards higher values in the sepsis group, although this did not reach statistical significance (Table 2).

Interestingly the proportion of patients under antibiotic treatment at the timepoint of enrolment was significantly higher in the inflammation group (44% vs. 18%), while after 24 h almost all patients of both groups (98%) received antibiotic therapy. The sources of infection of the septic group were classified as follows: Abdominal *n* = 20, pulmonary *n* = 9, catheter-associated *n* = 2, urologic *n* = 2, mediastinal *n* = 1. Of the sepsis group, *n* = 7 patients underwent surgery for focus elimination, *n* = 14 underwent an interventional procedure (e.g. drainage, endo-vac,…), *n* = 3 had catheters removed or changed and *n* = 10 had no intervention.

**Table 2:** Baseline characteristics at study inclusion (median, Min-Max)

	Inflammation (*n* = 25)	Sepsis (*n* = 34)	Student`s T-Test
Enrolment at POD (d)	6 (1–43)	7 (0–62)	ns
SOFA Score T0	**2 (1–13)**	**7 (1–18)**	*p* < 0.0001
Apache II Score T0	**12.0 (2.0–28.0)**	**18.5 (4.0–48.0)**	*p* < 0.01
Ventilation (NIV or INV) T0	*n* = 5 (20%)	*n* = 12 (35.3%)	ns
Vasoactive inotropic Score T0	**0 (0-23.3)**	**11.3 (0-98.3)**	*p* < 0.001
Temperature T0 (°C)	37.4 (35.2–40.1)	37.4 (35.8–40.0)	ns
Lactate T0 (mg/dl)	**9.8 (4.9-118.8)**	**18.8 (6.1-267.6)**	*p* < 0.01
Leukocytes T0 (/nl)	12.5 (4.1–29.4)	14.8 (1.3–62.8)	ns
Thrombocytes T0 (/nl)	323 (49–481)	220 (81–867)	ns
C-Reactive Protein T0 (mg/l)	126.8 (13.8-400.4)	179.7 (6.8-410.7)	ns
Procalcitonin T0 (ng/ml)	**1.09 (0.06–2.82)** (*n* = 8)	**6.69 (0.11–134.20)** (*n* = 24)	*p* < 0.05
Creatinine T0 (mg/dl)	**0.76 (0.35–5.64)**	**1.68 (0.47–4.78)**	*p* < 0.01
INR T0	1.11 (0.98–1.22)	1.15 (0.91–2.40)	ns
Albumin T0 (g/l)	31.8 (20.2–38.6)	29.3 (18.4–41.2)	ns
Antibiotics T0 n (%)	**11 (44%)**	**6 (18%)**	*p* < 0.05
Antibiotics T24 n (%)	24 (96%)	34 (100%)	ns

### Primary objective - SeptiScore ® results of sepsis vs. inflammation patients

The post-hoc diagnosis of the three experts categorized *n* = 25 (42.4%) patients as non-septic inflammation and *n* = 34 (57.6%) patients as septic. Overall *n* = 6 (10.2%) of the patients could not be primarily categorized and decision needed to be “forced”.

**Fig. 4:**
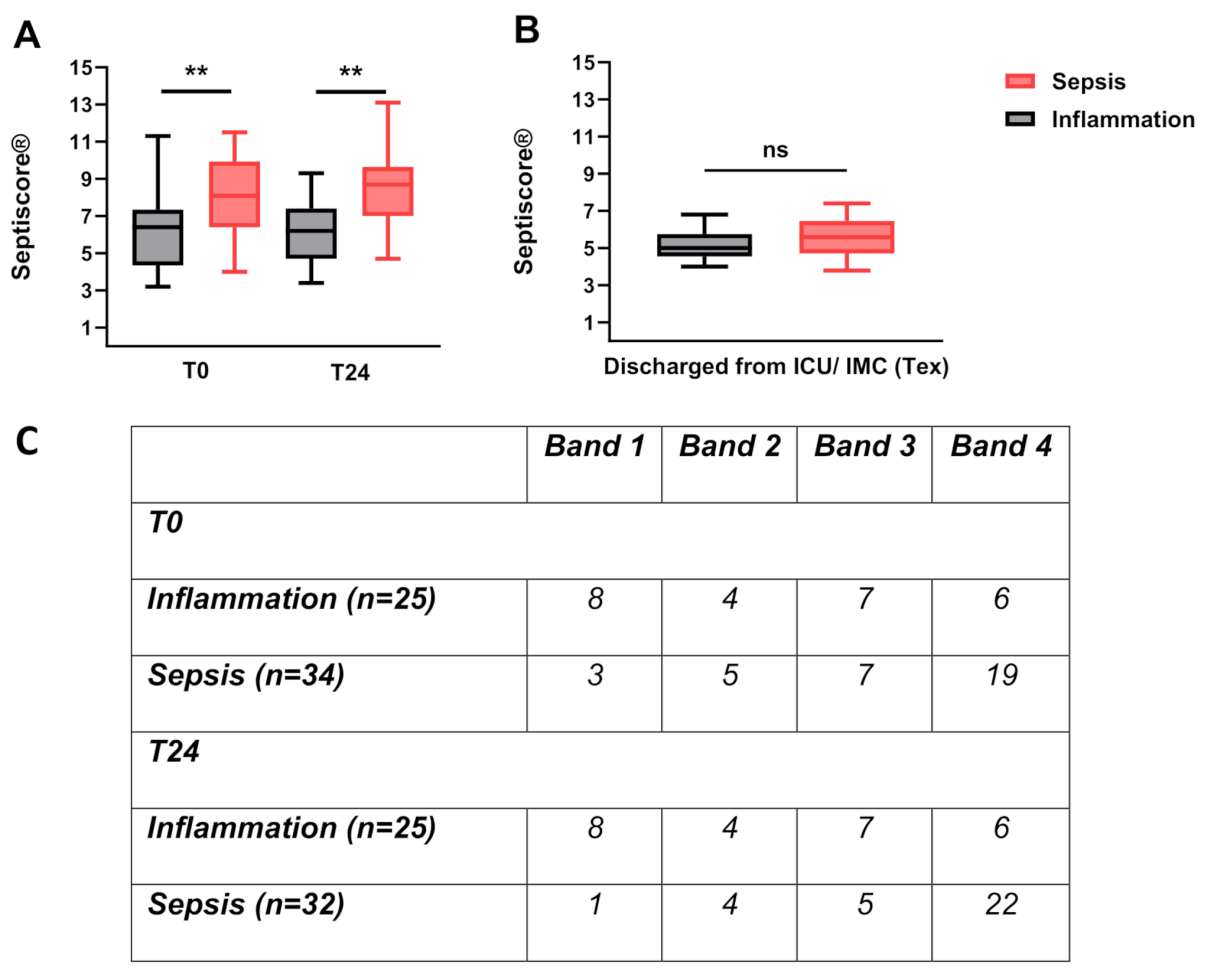
Comparison of SeptiScore® for sepsis and inflammation group The left Figure (**A**) shows the comparison of SeptiScores® for septic (“red”) and inflammatory (“black”) patients at the timepoints T0 and T24. For both measurements septic patients showed significantly higher (*p* < 0.01) mean SeptiScores®8.0 (± 2.2 SD) vs. 6.3 (± 2.1 SD) (T0) and 8.5 (± 2.1 SD) vs. 6.2 (± 1.8 SD) (T24). The right graph (**B**) shows the SeptiScore®of patients diagnosed with “Sepsis” (*n* = 20) and “Inflammation” (*n* = 17) at Tex, which does not show statistically significant differences. The sepsis group had a score of 5.6 ± 1.1 (Mean ± SD) and the inflammation Group 5.2 ± 0.8 (Mean ± SD) (*p* = 0.353). In **C** the distribution of patients in the different bands at T0 and T24 is shown. Mann Whitney Test, (*=*p* < 0.05, **=*p* < 0.01, ***=*p* < 0.001, ****=*p* < 0.0001)

Comparing T0 and T24 of the patients with the retrospective diagnosis of sepsis and the patients with inflammation, the SeptiScore® values showed significantly higher values in the mean (8.0/ 8.5 vs. 6.3/ 6.2) for the septic patients at both timepoints (Fig. 4).

There was a slight difference building the delta between T24 and T0, the patients in the inflammation group overall show a nearby stable situation (-0.1), while in the patients diagnosed as septic the SeptiScore® rose (+ 0.4) during the first 24 h after the beginning of treatment. Further there were 10 changes of one band to another, 5 patients increased and 5 patients decreased in the inflammation group between the first two measurements. Instead in the sepsis group 6 patients switched cumulatively 9 bands, all to higher bands and no one to a lower band. While at T0 23.5% (*n* = 8) of potentially septic patients had a SeptiScore® in Band 1 or 2 at T24 this were only 15.6% (*n* = 5). In addition, 4 patients of the sepsis group did not reach Band 4 at T0, but at T24.

Of the 6 patients, allocated to a group only after a forced retrospective diagnosis, 4 were categorized as inflammation and 2 as sepsis patients. Three of the 4 forced inflammation patients however showed SeptiScore® values in band 4 from 7.7 to 9.4 at both measurements (T0 and T24) while one had a SeptiScore® in band 3. One of the forced sepsis patients had a band 3 SeptiScore® at T0 increasing to a band 4 SeptiScore® at T24, while the other one had both T0 and T24 SeptiScore® results in band 1 as well as negative blood culture and other microbiological results.

Taken together only 1 of 6 patients with a forced diagnosis showed an aligned sepsis probability between the post-hoc expert diagnosis and the SeptiCyte® RAPID results.

At the timepoint of discharge from the high care facilities (ICU/ IMC) the SeptiScore® (Tex) decreased in both groups compared to the 24-hours measurement (T24) by 2.9 and 1.2 points in the in the sepsis group and the inflammation group, respectively (ns, *p* = 0.353). At this point 28 of the 37 Tex SeptiScore® values (75.7%) were situated in band 1 & 2. Regarding the 9 patients with band 3 or 4 Tex results, the analysis of all patients showed a weak negative correlation (r2=-0.44, Pearson) between the time passed since study inclusion and high SeptiScore®.

**Fig. 5:**
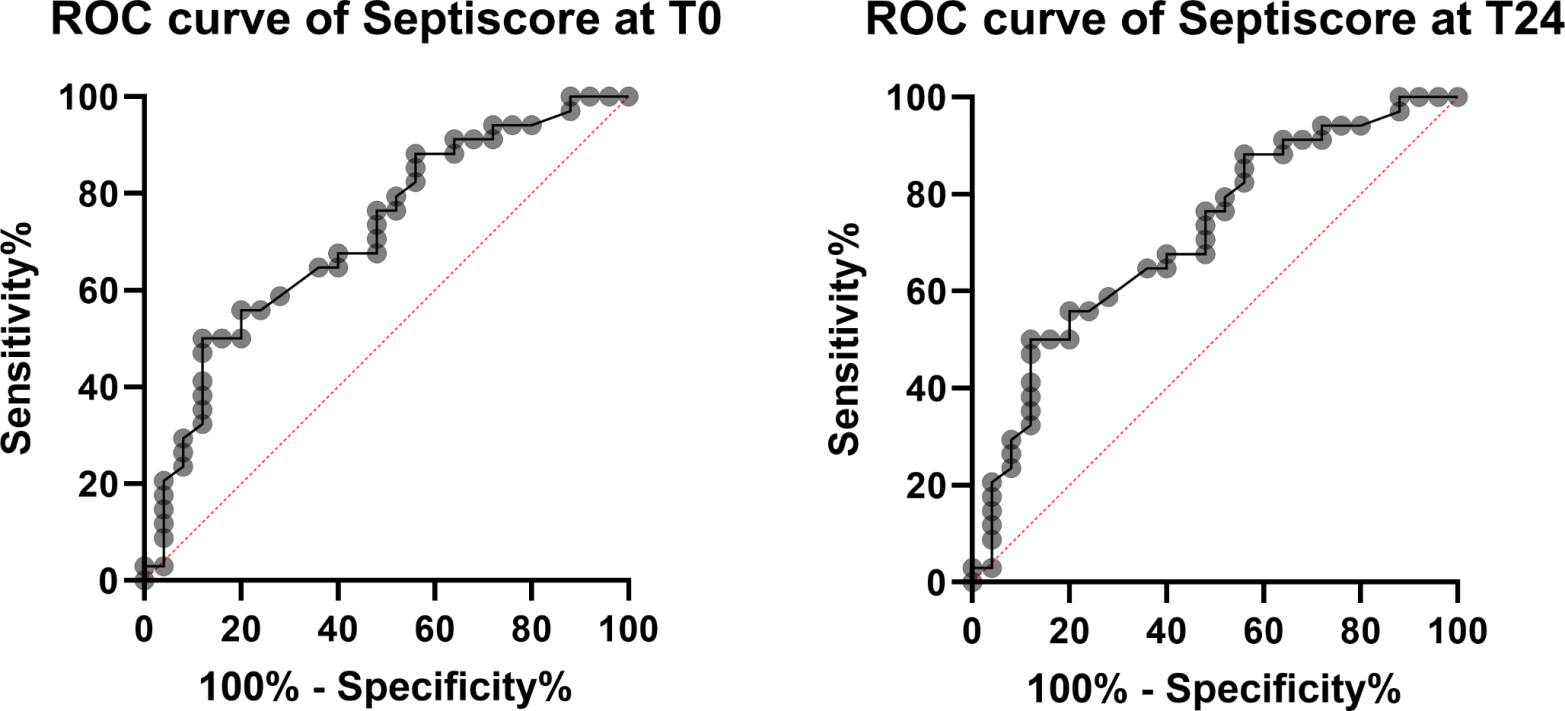
ROC analysis for the identification of “Sepsis” with the SeptiScore® The figures show the Receiver Operating Curves for the ability of SeptiScore® to discriminate between sepsis and inflammation according to the post-hoc diagnosis. In A T0 is shown and the AUC is 0.71 (CI 95% 0.58–0.85) (*n* = 59), while in B T24 with an AUC of 0.80 (CI 95% 0.68–0.91) (*n* = 57) is shown

Considering the ROC Analysis of T0 and T24 the SeptiScore® differentiated between sepsis and inflammation (Fig. 5). Excluding the patients with “forced” diagnosis enhanced the test performance with AUC T0 0.78 (CI 95% 0.65–0.91) (*n* = 53) and AUC T1 0.85 (CI 95% 0.75–0.96) (*n* = 51). The results shown in Fig. 5 are slightly better for the measurement 24 h after inclusion (T24) with an AUC of 0.80 (CI 95% 0.68–0.91) (*n* = 57). This effect of a better discrimination after 24 h is also shown by other inflammatory biomarkers. At T24 PCT of all available patients (*n* = 20) had an AUC of 0.83 (CI 95% 0.63-1.00) compared to T0 0.78 (CI 95% 0.62–0.95) (*n* = 32), the ones of C-reactive Protein and leucocytes were much smaller with 0.59 (*n* = 56) and 0.69 (*n* = 57) compared to 0.58 and 0.55 at T0 (*n* = 59).

### Secondary objectives - do the SeptiScore® results agree with the blood culture findings?

**Fig. 6:**
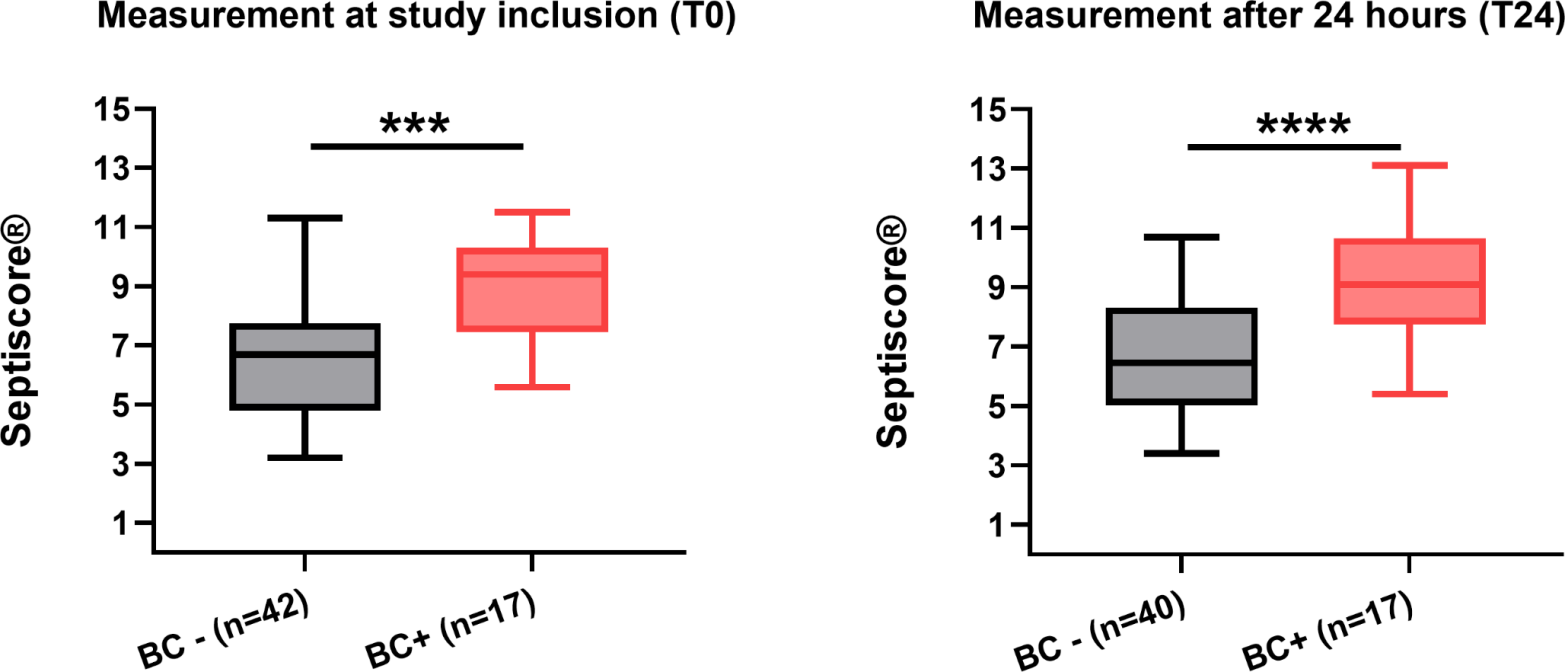
SeptiScore® comparison of blood culture positive to blood culture negative patients at T0 and T24 The SeptiScore®of patients with negative results of the blood culture drawn at study inclusion, showed significantly lower SeptiScore® values compared to patients with positive blood culture results at both timepoints T0 (left) 6.7 (3.2–11.3,*n* = 42) versus 9.4 (5.6–11.5,*n* = 17) and T24 (right) 6.5 (3.4–10.7,*n* = 40) versus 9.1 (5.4–13.1,*n* = 17) (Median and Min-Max). Mann Whitney Test, (*=*p* < 0.05, **=*p* < 0.01, ***=*p* < 0.001, ****=*p* < 0.0001)

Every Patient had at least one pair of blood cultures drawn at study inclusion (T0). Of these 17 (28.8% of all patients) had a microbiological finding. Most of the patients with positive blood cultures (BC+) had a SeptiScore® in Band 4 (82.4%) and none had a SeptiScore® in Band 1 (0%). Further 15/17 positive blood cultures were from patients in the sepsis group and only 2 were from patients in the inflammation group (both with SeptiScore® band 4 results). Comparing the SeptiScore® Results of the BC + and the BC- group, there are significantly higher SeptiScore®s in the BC + group (Fig. 6).

The discrimination of BC + patients seemed to work especially for blood cultures drawn simultaneously with SeptiCyte® RAPID measurements. Positive blood cultures drawn at timepoints different than the SeptiCyte® RAPID measurements (*n* = 9) showed significantly lower SeptiScore®s than the ones taken at the same time (*n* = 17) as the SeptiCyte® RAPID measurements (6 (4.0-11.3) vs. 9.4 (5.6–11.5), Median, Min-Max) (*p* < 0.05).

A comparison of various laboratory parameters and scores for the differentiation of positive and negative blood culture results revealed that both the SeptiScore® measurements at T0 and T24 and the PCT had the highest AUC values, followed by CRP and leukocytes. In contrast, disease severity scores such as SOFA and APACHE-II score demonstrated poor discrimination between BC + and BC- patients (Table 3). Nevertheless, the discriminatory power of Septiscore(r) reported as ROC analysis AUC increased in combination with other parameters such as PCT (0.94 CI95% 0.875–0.996) as well as SOFA score (0.82 CI95% 0.71–0.92) or CRP (0.74 CI95% 0.62–0.87).

**Table 3:** AUC of different laboratory inflammation markers and Disease Severity scores *negative vs. positive blood culture results*

	T0	T24
SeptiScore®	*AUC 0.79* *(CI 95% 0.66–0.91) (n = 59)*	*AUC 0.83* *(CI 95% 0.72–0.94) (n = 57)*
PCT	*AUC 0.71* *(CI 95% 0.52–0.90) (n = 32)*	*AUC 0.73* *(CI 95% 0.46-1.00) (n = 20)*
CRP	*AUC 0.67* *(CI 95% 0.52–0.82) (n = 59)*	*AUC 0.64* *(CI 95% 0.48–0.80) (n = 56)*
Leukocytes	*AUC 0.64* *(CI 95% 0.46–0.83) (n = 59)*	*AUC 0.50* *(CI 95% 0.31–0.69) (n = 57)*
Apache II	*AUC 0.66* *(CI 95% 0.51–0.81) (n = 59)*	*AUC 0.63* *(CI 95% 0.47–0.79) (n = 57)*
SOFA Score	*AUC 0.67* *(CI 95% 0.51–0.82) (n = 59)*	*AUC 0.68* *(CI 95% 0.53–0.83) (n = 57)*

The different pathogens that were detected in BC + and the corresponding SeptiScore®s are shown in Table 4, in some of the patients more than one pathogen had been proven.

**Table 4:** Differentiation of pathogens found in positive blood cultures and corresponding SeptiScore® at T0 and T24

Pathogen	T0	T24
E. coli (*n* = 5)	8.5 (± 1.0)	9.1 (± 1.8)
Candida albicans (*n* = 3)	9.4 (± 1.7)	9.7 (± 3.1)
Staph. epidermidis (*n* = 3)	7.3 (± 1.9)	8.6 (± 1.2)
Strept. anginosus (*n* = 2)	7.5 (± 2.5)	7.4 (± 2.8)
Enterobacter cloacae (*n* = 1)	10.2	9.9
Klebsiella pneumoniae (*n* = 1)	10.4	9.9
Klebsiella oxytoca (*n* = 1)	11.5	12.0
Serratia marescens (*n* = 1)	10.9	11.4
Staphylococcus hominis (*n* = 1)	6.7	7.3

### Secondary endpoints - do other microbiological findings or antibiotics have an influence on SeptiCyte® RAPID results?

**Fig. 7:**
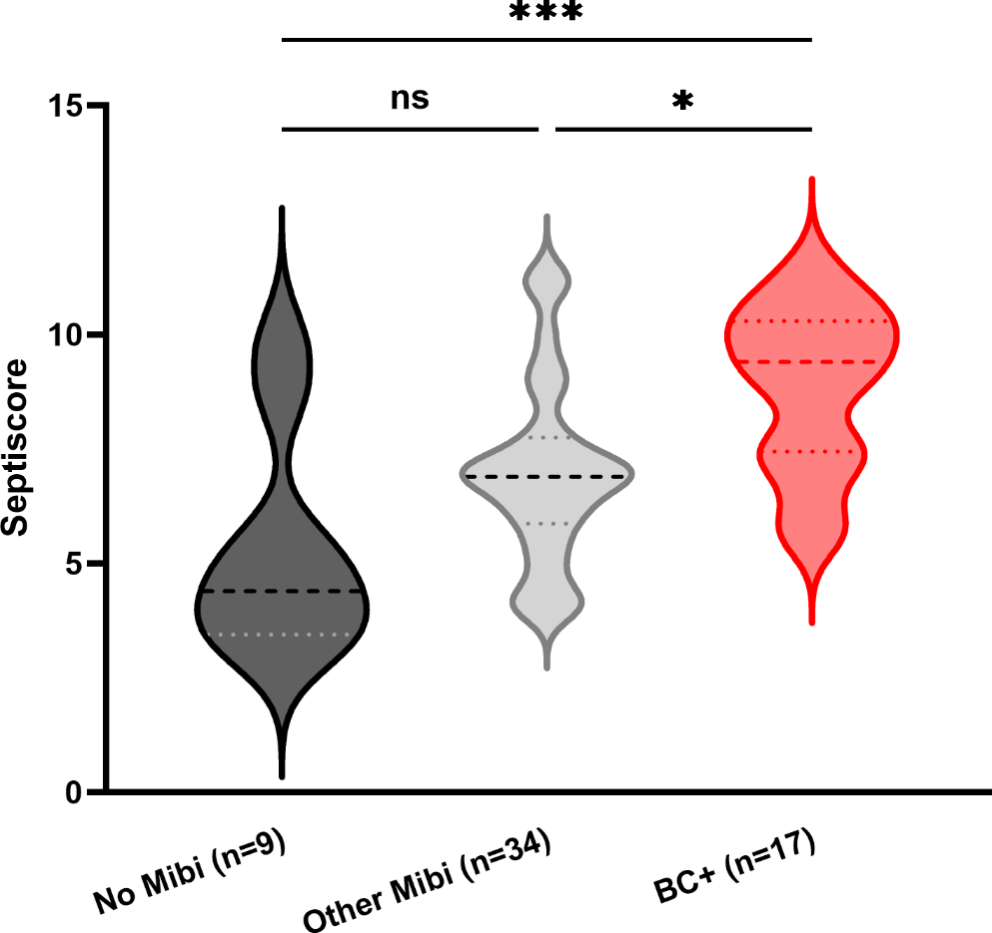
Comparison of SeptiScore®s at T0 for patients with different microbiological findings The graph shows SeptiScore®s (T0) rising in accordance with positive microbiological findings. From left to right patients without any microbiological finding during the study period (*n* = 9, 4.4 (3.2–9.9), Median (Min-Max)), patients with different positive microbiological findings but a negative blood culture at admission (*n* = 34, 6.9 (4.0-11.3), Median (Min-Max)) and patients with a positive blood culture at T0 (*n* = 17, 9.4 (5.6–11.5), Median (Min-Max)) are shown respectively. Kruskal-Wallis Test, (*=*p* < 0.05, **=*p* < 0.01, ***=*p* < 0.001, ****=*p* < 0.0001)

The 59 study patients were divided into three groups. The “No Mibi” Patients (*n* = 9, 15%) had no pathogen detection during the study observation period (-7 days before Inclusion until 6 weeks after inclusion). The “Other Mibi” Group (*n* = 33, 56%), contains all the patients which had positive microbiological findings other than the blood cultures at study inclusion while the “BC + T0” Group (*n* = 17, 29%) consists of all the patients which had positive blood cultures at study inclusion regardless of whether further positive microbiological results were obtained. Figure 7 shows higher SeptiScore® values for other Mibi patients while the highest SeptiScore®s are obtained in BC + T0 Group.

Separating these three groups by the retrospective diagnosis “Sepsis” vs. “Inflammation” showed the same trend of increasing SeptiScore®s for the BC + T0 patients. Patients categorized with inflammation had SeptiScores® with the following means (± SD): No Mibi, 4.2 (± 0.88), *n* = 7 (28%); Other Mibi, 6.9 (± 1.89), *n* = 16 (64%); and BC + at T0, 8.4 (± 1.41), *n* = 2 (8%). In the sepsis group, only two individuals had no microbiological results but presented with a high SeptiScore® (mean 9.3 ± 0.84), and one of these patients died within 24 h. The other two groups, Other Mibi and BC + T0, exhibited a similar SeptiScore®s mean (± SD) trend as previously described: Other Mibi 7.1 (2.2) *n* = 17 (50%) and BC + T0 8.9 (1.88) *n* = 15 (44.1%).

In total *n* = 58 (98%) of all study patients received extended antibiotic therapy for various reasons during their clinical stay. Further, all patients were divided into a group which was under antibiotic treatment already at admission (*n* = 17) and a second one which had no antibiotic therapy at T0 (*n* = 42). The SeptiScore® results median (Min-Max) at T0 were 7.0 (3.2–11.5) without previous antibiotic therapy and 7.4 (4.4–11.1) under antibiotic therapy. At T24 98% of patients were under antibiotic therapy. The SeptiScore®s median (Min-Max) remained similar between the groups, 7.5 (3.4–13.1) vs. 7.3 (4.7–10.7) with no statistically significant difference at neither timepoint.

## Discussion

The prospective observational SeptAsTERS study evaluated the SeptiCyte® RAPID test in a postoperative cohort after major abdominal surgery. The study found that SeptiCyte® RAPID could distinguish between patients with postoperative clinical deterioration due to inflammation versus sepsis. As such, the test results aligned with the post-hoc diagnosis evaluation by blinded experts.

While a previous pilot study with the predecessor SeptiCyte® LAB test suggested its potential to diagnose infection in postoperative patients [19], this study represents the first evaluation of SeptiCyte® RAPID in a strictly surgical cohort. The results of SeptAsTERS demonstrated high AUCs in major abdominal surgery patients when referred to a high-level ICU (T0) due to clinical deterioration as well as within the first 24 h. Previous analyses indicated that the test is not impacted by medications like corticosteroids and immunosuppressants [20]. In this analysis, SeptiScores® were similar with or without antibiotic therapy, providing first hints, that antibiotic therapy during the first 24 h does not compromise the test performance.

The results of SeptAsTERS demonstrated higher accuracy of SeptiCyte® RAPID compared to CRP alone. The AUC was somewhat lower than a comparable SeptiCyte® RAPID validation in a non-strictly surgical cohort (AUC 0.807–0.820) [12, 21–24]. Early sepsis diagnosis has been shown to improve survival and reduce treatment costs [25–27]. Despite considerable innovation, the results of routine diagnostic microbiology including newer technologies like PCR or NGS often need many hours until getting a result [28, 29]. The „hands-on“ time for blood collection and pipetting takes less than < 10 min and the subsequent measurement of the SeptiCyte® RAPID test up to obtaining the result takes approximately 60 min. As such, the availability of test results within the critical timeframe of sepsis care in approximately 1 h after running the test point-of-care by ICU staff may be seen as advantageous.

Excluding sepsis could further reduce unnecessary antibiotic use and associated adverse effects [30]. Serial SeptiCyte® RAPID testing may offer advantages, as at least 4 patients had scores in Band 4 only on the 24-hour repeated measurement, which otherwise may have been missed. But while the goal of reducing unnecessary antibiotic use is important, the benefits of SeptiCyte® RAPID must be carefully weighed against its diagnostic limitations and the uncertainties surrounding sepsis diagnosis.

Furthermore, in the present study the SeptiCyte® RAPID test was also compared with the results of microbiological findings. In general, the presence of a positive microbiological finding showed a tendency towards higher SeptiScore® values. Especially patients with simultaneously taken positive blood cultures had significantly higher SeptiScore® values. Due to the small number of cases, no differences in the scores for specific pathogens could however be determined. Although blood cultures are the gold standard to diagnose blood stream infections, they only detect pathogens in 30–50% of suspected sepsis cases [31] and can be falsely negative due to antibiotic therapy [32–34]. This is consistent with the results of the present study in which 17 patients had positive blood cultures at admission, respectively in 44% of all sepsis patients and 8% of systemic inflammation patients. All sepsis patients with positive admission blood cultures had high, band 4 SeptiScore® results. High SeptiScore®in patients with suspected infection could therefore suggest the intensive collection of microbiological samples, while a low SeptiScore® value can help to recognize a positive blood culture as contamination and avoid the negative medical and monetary consequences of false positive blood cultures [35]. The potential application of SeptiScore® to increase the pre-test probability for advanced diagnostic tools, such as next-generation sequencing or reduce the occurrence of ambigugous results as well as avoid unnecessary costs is intriguing but requires further research to better understand the clinical interpretation and cost-effectiveness [36].

A key strength of our study lies in the rigorous execution of a thorough protocol in a strictly surgical population. However, there are important limitations of our findings. These include the exploratory nature of the study as well as a relatively small, but strictly selected collective. Further the PCT was missing in approximately half of the study population and overrepresented in the sepsis group. These aspects may have had a confounding effect on statistics. However, pretest probability is relatively high as the patients were recruited in a large centre with a high number of cases with abdominal sepsis.

Furthermore, in this highly complex patient population, SeptiCyte® RAPID, showed difficulties particularly in differentiating borderline cases in Band 2 and 3, this could be a potential limitation in its clinical application. But as reported also the retrospective diagnosis by experts was “unanimous” in only 37.3% of patients, “consensus” of 2 experts only in 52.5% of cases and had to be forced in 10.2% of patients. Especially in the 6 “forced” cases there was an excessive discrepancy between retrospective diagnosis and SeptiCyte® RAPID in 5/6 (83.3%) patients. Although the number of “forced” patients is consistent with other studies that have used this method (9.8% in [12]), it highlights the difficulty of making the diagnosis “Sepsis” in borderline cases and points to a high level of remaining uncertainty. While inflammation was more often diagnosed unanimously, septic patients were more likely to be diagnosed by consensus, which seems to be more difficult. The procedure of post-hoc diagnosis may have had an influence on the evaluation of the SeptiCyte® RAPID test, as it has its own limitations.

In total SeptiCyte® RAPID classified 10–15% of patients inconsistently compared to the retrospective expert diagnosis (Band 4 for inflammation or Band 1 for sepsis). In “unanimous” cases (*n* = 22), there was a discrepancy in 4 (18.2%) patients and in 16 (72.7%) the diagnosis agreed, which is significant given the high mortality of sepsis and should be taken into consideration.

In our study, SeptiCyte® RAPID in comparison with PCT alone had slightly higher AUC values, suggesting a non-inferiority to this established biomarker which is used also for guiding antibiotic therapy [21]. Nevertheless, factors like surgery or transfusions can lead to PCT elevation in the perioperative context [22, 23] and a study in critically ill postoperative abdominal surgery patients found PCT could not accurately predict antibiotic treatment response [24]. By combining SeptiCyte® RAPID and PCT the discriminating power rises over the single AUC`s as already shown in previous studies [12–14]. This indicates that at the moment, all available clinical parameters and diagnostics should be embedded in a combined diagnostic algorithm to reach a maximum of diagnostic accuracy.

Considering the limitations of this pilot study, our results are promising, seeing our results reproduced in a larger cohort of patients with suspected infection following major surgery or trauma is a next logical step.

## Conclusion

The SeptAsTERS study is the first evaluation assessing the performance of SeptiCyte® RAPID for point-of-care molecular host response analysis in patients with clinical deterioration due to suspected infection after major abdominal surgery. Using gene expression signatures to detect sepsis is a novel approach for early diagnosis which is not yet incorporated into clinical routine. The point-of-care molecular host response assay was able to differentiate between sepsis and non-septic sterile systemic inflammation. Further, especially patients with positive blood cultures could be recognised by host response analysis without observed influence of previous antibiotic therapy. However, the limitations of this technology, especially when compared to existing biomarkers and diagnostic methods, should be carefully considered in interpreting the results. To ensure accurate diagnosis, the SeptiCyte® RAPID test needs to be embedded in a comprehensive diagnostic algorithm that includes clinical examination, biomarkers, and microbiological findings. Future studies should focus on the investigation of molecular host response-driven risk-stratified antimicrobial therapy to enable early antibiotic treatment in sepsis and to avoid antibiotic overuse in sterile systemic inflammation.

## Data Availability

Data is provided within the manuscript or supplementary information files. Further data are made available on reasonable request through the principal investigators.
